# Dried Fruit

**Published:** 1873-04

**Authors:** 


### DRIED FRUIT.

Millions of bushels of apples and pears and peaches are rotted and otherwise wasted every year, which, if promptly gathered and cared for and dried, could be used and would make healthful and nourishing food. That man will be a benefactor to his race who will discover some handy, inexpensive method by which every farmer could dry his own fruit; not only would millions of money be saved thereby, but an immense amount would be added to the food supply of the world. Dried fruits are next in naturalness to the green and ripe, because they contain all their constituents except the watery portion, which can be easily added when about to be prepared for table use. The most healthful and nutritious articles of food in the world are its grains and its fruits. The grains make blood which gives warmth and strength, the fruits keep it pure by their opening effect on the system. It is troublesome and costly to preserve or can fruits, and their transportation is dangerous from breakage, and expensive, but fruit can be dried so that it will not decay, and can be transported as easily as corn and wheat, and pickled and cured meats.

Every family, as a means of health, should have fruit in abundance in some form or other on the table, at least once every day. Alden, of Delaware, and Gould, of California, both claim inexpensive and convenient methods of fruit drying and have patented them; how available they are is not known, but agriculturists everywhere should make a note of it.

———o———

Mr. Vick, of Rochester, N. Y., the great seedsman, florist and nurseryman, has the reputation of having the most beautiful and extensive assortment of roots and flowers in the whole United States.

